# Trust me, I’m a Doctor: Strategies for Combating Imposter Feelings Among Physicians and Medical Students

**DOI:** 10.1007/s40670-025-02283-1

**Published:** 2025-01-24

**Authors:** Krystal Rampersad, Rachqueda Salfarlie, Arlette Herry, Michael Montalbano

**Affiliations:** https://ror.org/01m1s6313 grid.412748.c St. George’s University, True Blue, St. George, Grenada

**Keywords:** Self-concept, Medical education, Psychological stress, Dread, Tenacity

## Abstract

Imposter phenomenon, characterized by persistent self-doubt and a fear of being exposed as a fraud, has become increasingly recognized within the last decade. Recent studies show that imposter phenomenon is widespread within the medical community, yet there exists no consensus framework or model to understand and assist those experiencing the phenomenon. This article aims to provide a practical guideline for managing imposter phenomenon in physicians and medical students at all levels. We propose that the use of specific strategies designed to support medical professionals can aid in recognizing, confronting, and overcoming imposter feelings.

## Introduction

Imposter phenomenon, also known as imposter syndrome, was first coined in 1978 to describe the internalized experience of believing that an individual is not as competent as others perceive them to be, often despite the individual’s obvious achievement and success [1]. Although both terms are often used interchangeably, impostor phenomenon may be the more appropriate term since the state is not recognized as a clinical diagnosis but as a cultural occurrence. While imposter phenomenon (IP) was initially noted in select groups with a specific focus on women, recent studies have shown it to be prevalent among both men and women as well as across various cultures [2]. Across the different groups experiencing impostor phenomenon, there are similar cognitive distortions including attributing success to luck or coincidence, shying away from praise, downplaying the significance of accomplishments, and, arguably most significant, the enduring self-doubt concerning one’s competence and skills [3].

**Fig. 1:**
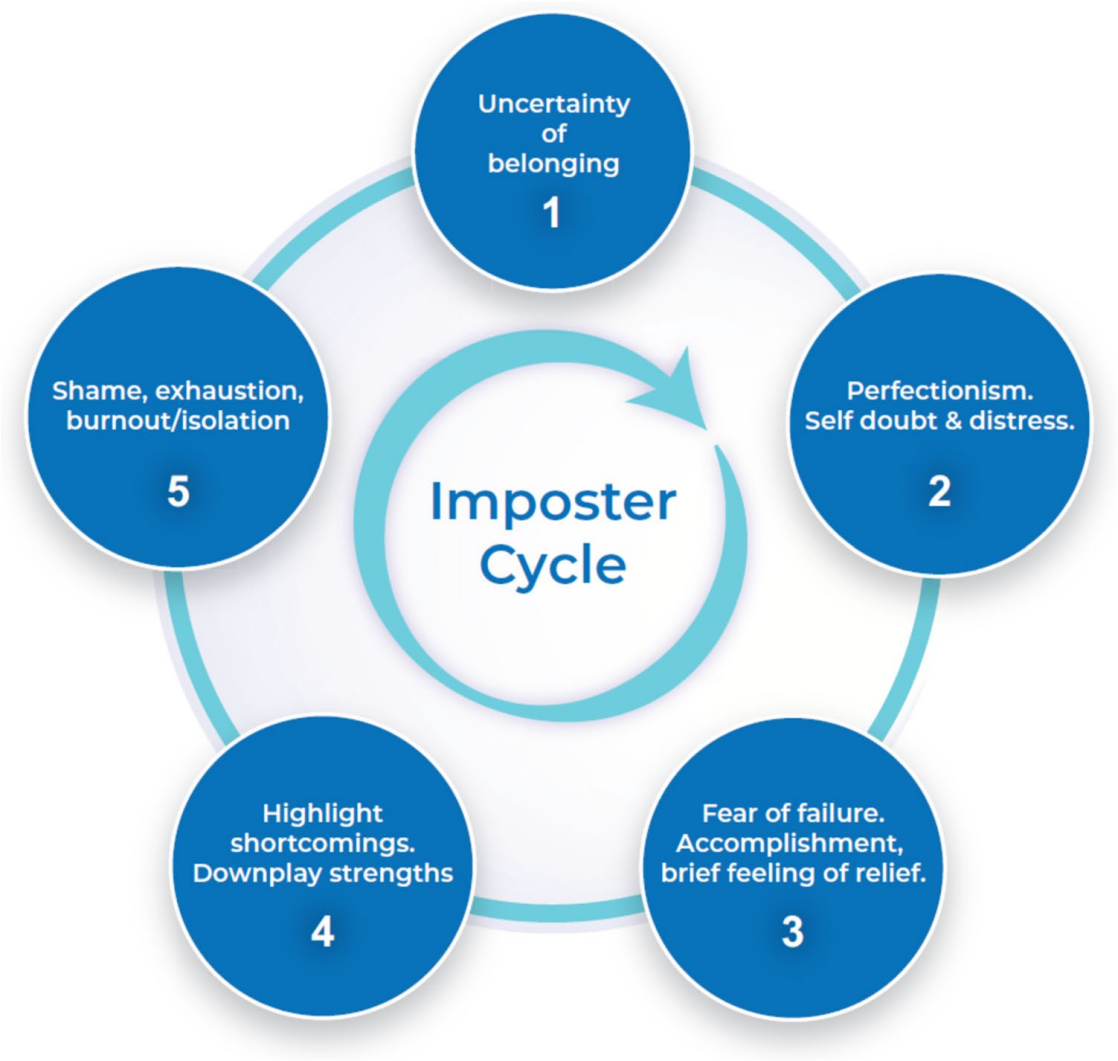
The IP cycle, for which management strategies can be implemented at various stages

As individuals experiencing IP are more likely to experience feelings of guilt, anxiety, and diminished self-esteem, IP can become a significant barrier to an individual’s mental health, career trajectory, and overall state of well-being [2]. Since Clance et al.’s initial publication, IP has notably proliferated across scientific literature, particularly within the last decade. Recent studies have shown that IP can particularly affect people in high-achieving fields, such as medicine [4, 5]. This is especially concerning for medical professionals since the aforementioned factors, combined with the drive to professionally succeed and the many stressors present within the medical profession, can have a profound impact on physicians. This in turn can negatively affect physician mental health and well-being, as studies using the Maslach Burnout Inventory have found statistically significant associations between burnout and IP [5, 6]. These factors in combination lead to a pattern where the individual perpetuates the concept of oneself as a fraud, which further propagates IP cycles (see Fig. 1). These elements reinforce the idea that the intense and high-stress conditions, found in both medical education and healthcare practice, can intensify or reveal IP in vulnerable individuals.

To the best of our knowledge, there is currently no formal consensus on a medical definition or diagnostic gold standard for IP. However, certain aspects identified by Clance have been developed and noted in literature published within the last decade. Considering the significant impact that IP can have on the mental health, well-being and career trajectory of medical professionals, as well as the larger impact on the healthcare system, it is noteworthy that the literature does not contain clear, practical examples of how IP can be managed. To address this, we share five specific techniques that can be used to manage imposter phenomenon in medical professionals. We hope that these techniques can empower physicians and medical students to overcome self-doubt and build confidence in their professional capabilities, ultimately enhancing both their well-being and the quality of care that is provided.

### Encourage Open Discussion of Imposter Phenomenon and Inclusive Environments

Comparing one’s own experiences and struggles to another’s accomplishments can accentuate feelings of inadequacy and exacerbate symptoms of IP, as well as impact both peer relationships and relationships among multi-disciplinary team members. Therefore, hearing that successful physicians, who have achieved notable accomplishments, have also confronted and successfully navigated similar feelings can be incredibly reassuring as well as offer validation and inspiration to individuals grappling with their insecurities. Openly discussing IP as a common experience that affects many people, regardless of their level of success or competence, can help individuals realize they are not alone and that subjective feelings are not indicative of objective incompetence. By creating a safe environment that encourages open exchange, communication, and learning, medical professionals may be less reluctant to share their own experiences [7].

Leaders and mentors should share their experiences with imposter feelings when appropriate. Since many people with IP are afraid of admitting their shortcomings, usually due to the belief that others will see them the way they see themselves, open dialogue facilitated by senior faculty can create healthier expectations and a culture where mistakes are not seen as failures but as a learning experience. Such discussions can normalize the concept of IP and encourage individuals to seek support, lead to the development of coping strategies, and cultivate healthier self-perceptions [8]. Forming a healthy self-perception in medical training can be especially difficult due to the high expectations placed on medical students and physicians. Additionally, the environment in which medical education and training takes place is often highly competitive as learners’ skills, knowledge, and competencies are constantly assessed [9]. Therefore, discourse on IP during orientations, onboarding, and development sessions may abate the development of these feelings during later medical training [7]. Providing avenues such as access to therapy and resilience workshops with the specific aim of targeting IP can also play a pivotal role in diminishing its prevalence within the medical community.

In scientific careers, a sense of professional inadequacy often develops that forces individuals to question their sense of belonging, regardless of their level of skill or knowledge [10]. As this uncertainty of belonging is intricately linked to IP, it is imperative to create inclusive environments in medical schools and clinical settings that foster a sense of belonging. Embracing diverse backgrounds, perspectives, and learning styles helps alleviate IP while also promoting creativity, innovation, and courage [11]. Inclusive environments can mitigate IP in a variety of ways. First, individuals feel valued and accepted, which reduces the isolation often associated with IP. When medical professionals see themselves represented among peers, leaders, and faculty, it reinforces their sense of belonging. Second, connecting with mentors who share similar backgrounds or experiences can help combat feelings of inadequacy. Third, actively seeking and valuing diverse perspectives and ideas reinforces the importance of everyone’s input, helping individuals recognize the value of their unique contributions. Fourth, implementing institutional policies that promote supportive environments, work-life balance, and well-being can reduce stress and burnout, which often exacerbate IP [12, 13].

### Set Realistic Expectations

Perfectionism, as identified by Clance et al., is defined by an overarching “need to be the best.” [1]. This trait spans a spectrum of behaviors driven by self-imposed, practically unachievable standards and goals, common among individuals with IP [14]. We agree with Rehsi and McCarthy (2023) that the competitive and hierarchical nature of the medical profession sends a mixed message to healthcare professionals who, under supererogatory expectations and limited by both time constraints and heavy demands, often experience confusion and frustration at being unable to fulfill incongruent factors [15]. Unattainable benchmarks fuel a harmful feedback loop, pushing those affected to continually strive to “be the best” despite the detriment to their well-being. Thus, it is important to help individuals set achievable goals and let go of the unattainable concept of perfection. Emphasizing the value of progress over perfection, and the importance of learning from mistakes, should reduce feelings of IP.

Medical education operates within a uniquely high-stakes environment where maintaining rigorous standards is essential to prepare students for the various responsibilities they will assume as healthcare professionals. Future physicians must receive training not just in knowledge acquisition, but also in the clinical skills and emotional resilience necessary for high-stress, real-world scenarios [16]. Therefore, while stress reduction is important for student well-being, it must be balanced against the recognition of the stresses inherent in medicine to achieve the standards required to ensure clinical competence and patient safety. Leaders in medical education, such as program directors and administrators, should carefully consider how to support students without compromising these vital standards. Recognizing the diverse challenges students face, medical education programs can benefit from dynamic support systems. These systems should address a range of student experiences, from high achievers who feel intense pressure, to those struggling with the core competencies of their training and may be experiencing feelings of inadequacy.

### Do not be Afraid of Failure or Success

Individuals grappling with IP often downplay their talents and subsequently suffer large losses in self-esteem when faced with failure [17]. Professionals with IP are more likely to be stuck in a cycle where an underdeveloped ability to appropriately recognize and accept success may cause them to be confused when their accomplishments lead to elevated expectations or workloads. This in turn can result in increased feelings of anxiety due to an inability to accept success and increased feelings of imposterism as the individual expects that greater responsibility will reveal them as deficient [18]. Changing norms around the use of social media in recent years may have also heightened feelings of inadequacy among medical students, who are frequently exposed to the achievements of their peers. In the competitive field of medicine, where advancement is often defined by successful performances and impressive outcomes, consistent comparisons can contribute to imposter phenomenon and mental burnout. The meticulously curated images on social media can blur the line between reality and perception, intensifying self-doubt [19]. However, true success in healthcare is not solely about grades or recognition. Emphasizing health, personal growth, and a balanced lifestyle can help students manage expectations and reduce unnecessary pressure against unrealistic standards.

People with IP are more likely to feel extreme embarrassment and humiliation if they perform below average. To mitigate the impact of IP and encourage a healthier, more balanced professional identity, medical professionals should be encouraged to acknowledge that failure is part of the learning cycle, that some amount of imperfection is inevitable, and recognition coupled with acceptance of this can lead to improved levels of confidence. For those with IP, feelings of anxiety are often felt when approaching a task which can trigger behaviors such as procrastinating or over-preparing. Those who procrastinate continue to see themselves as fraudulent, believing that they successfully tricked people into thinking they were competent. Conversely, with overpreparation, they may start to believe that more effort than others is needed to succeed, thereby reinforcing imposter feelings [20, 21]. After completing a task, individuals with IP feel brief relief which is quickly followed by worry. They fear that their achievements will raise others’ expectations which they believe they cannot meet, or that their work is subpar and will expose them as the fraud they believe they are [21]. Therefore, amplifying the good and celebrating achievements among healthcare workers can help to manage IP [22]. By encouraging medical professionals to focus on and celebrate achievements, they may be more likely to internalize positive feelings. Recognizing accomplishments also helps build self-confidence and counter feelings of fraudulence. These achievements may not necessarily require a significant impact. Any achievement should be included such as publications, awards, or even a thank you e-mail from a patient or colleague. Individuals should be encouraged to keep track of and celebrate their achievements, however small, as minor victories can be just as meaningful as major ones.

### Emphasize Coping Strategies, Perseverance, and a Growth Mindset

IP is associated with cognitive distortions that reinforce irrational thoughts and beliefs [23]. As such, cognitive-behavioral techniques including cognitive restructuring, mindfulness, and journaling may help affected individuals manage feelings of perceived inadequacies, self-doubt, and anxiety. Cognitive restructuring involves reframing negative thoughts and replacing them with realistic, balanced, and precise ones; while mindfulness encourages individuals to observe thoughts and emotions without passing judgment and allows for detachment from negative self-talk and self-criticism, thus creating a space of unbiased self-reflection [24]. Additionally, journaling also proves to be a powerful tool in overcoming IP. By putting thoughts on paper, individuals identify and gain clarity and insight into their emotions, thus enabling them to recognize thought patterns that can hinder growth. For example, writing about achievements, strengths, experiences, and goals can serve as reality checks against catastrophizing and unrealistic expectations. Furthermore, these coping strategies can be practiced and honed within individual or group-based settings that allow one to feel supported while building self-efficacy [25].

It is also important to emphasize deliberate choices in the language used when referring to self-advocacy. This means avoiding restrictive modifiers such as “just” and “only” when talking about one’s contributions as well as not over-apologizing for any flaws, whether real or perceived. In a profession where medical students and doctors often face obstacles like struggling to master a clinical technique, poor academic performance, and dealing with difficult patient interactions, a greater failure lies in abandonment rather than perseverance. While these strategies take time and often require social support, they can serve to eliminate stressors, build confidence, and reduce both emotional exhaustion and IP [26]. Over time, with a sufficiently positive mindset, one may even perceive these same thoughts and feelings positively as an urge for improvement.

For those with IP, it is often difficult to deal with the fear of making mistakes and associated psychological concerns [27]. Those with IP also show a tendency to focus on the negative aspects of feedback after completing a task, which is often linked to traits of a fixed mindset. Since mindsets can impact beliefs and behaviors, changing the traits that facilitate feelings of inadequacy has been shown to assist students struggling with depression [28]. Wolcott et al. [6] identified key traits that should be modified: cultivating intellect, perseverance, optimism, and using feedback for learning. Adopting these traits to address IP can help reframe negative feedback and allow setbacks to be seen as opportunities to grow and improve. This approach also promotes a growth mindset by redirecting attention from a fixed perspective to one that emphasizes evolution and effort [29]. Medical professionals who adopt this mindset are more likely to adjust to new knowledge, procedures, and practices with ease, dispelling the notion that the medical field is only for high achievers. In the medical field, where professionals may doubt their intelligence and abilities, emphasizing progressive development of skills through hard work can foster continuous learning while encouraging persistence and resilience to help individuals overcome self-doubt. Promoting a growth mindset requires not only changing the language and communication approach, it also requires having instructional support as well as incorporating teaching methodologies [30].

Determination and trust in the system in which one was placed have been shown to be effective in combating imposter thoughts [10]. To specifically address this, putting confidence in the judgments of those who admitted them to medical school and conferred their MD has been shown to be helpful. Instead of questioning these decisions and succumbing to self-doubt, adopting the perspective that these experts recognized their potential may be more beneficial. This can help minimize some of the pressure those with IP feel as they navigate the challenges of medical training. By reframing self-doubt as a reminder of the confidence others have in their achievements and abilities, individuals may find relief from some of the pressures associated with IP. This shift in perspective encourages resilience and allows them to focus on growth rather than on questioning their worthiness in the field.

### Actively Seek Support Through Peers, Mentorship, or Asking for Help

As competition becomes more commonplace between medical practitioners, it should be expected that IP positively correlates with levels of emotional exhaustion at work and negatively correlates with career planning, career striving, and motivation to lead [26]. One way to oppose these negative trends is to maintain a focus on shared goals such as improved health outcomes in patient care. Joint pursuit of these shared goals can be facilitated by peer support groups where colleagues can gather to offer a supportive atmosphere for constructive feedback, reassurance, and exploration of avenues that may normalize feelings related to IP [31]. These common experiences in turn have been found to make significant differences in preserving work ethic and guarding against emotional exhaustion [26]. Encouraging open dialogue between peers is likely to reinforce the concept of growth and learning as well as allow those dealing with imposter feelings to be included and understood.

In IP, several negative associations have been identified, particularly with respect to career and personal well-being such as self-efficacy beliefs and job satisfaction. In combination with decreased motivation for career planning and the heavy demands placed on medical professionals, this may lead to burnout over time [32]. Through coaching and mentoring, experienced professionals can offer guidance, advice, and reassurance to medical professionals struggling with IP. Coaching initiatives serve to enhance participants’ comfort with uncertainty, boost their confidence in problem-solving through collaboration, and encourage an adaptive approach [33]. Coaching sessions can therefore create a supportive environment where IP feelings can be diminished through encouragement and constructive feedback, ultimately leading to improved performance, learning, and growth [34]. While in supportive environments, mentorship can help address and provide strategies to combat IP, from admitting imperfections, to focusing on strengths, and even through discussions that provide specific feedback on performance since avoiding feedback may exacerbate imposter feelings by making individuals assume the worst about their performance or ability [35]. Coaching and mentorship offer a safe and nurturing environment for medical professionals, enabling them to confront feelings of inadequacy and combat negative thoughts associated with IP [36].

Within the culture of medicine, seeking help is sometimes seen as an admission of weakness or lack of competency. During times of transformation or transition, IP has been shown to become more prominent [37]. This can significantly impact medical students and junior physicians as they navigate new responsibilities and environments. Awareness of this and other stressors can help individuals recognize when they need additional support, allowing them to seek help instead of isolating themselves in times of need. Professional growth requires not only repetition but an eventual transformation to an improved state [38]. In this way, being aware of one’s limitations and acting accordingly is a strength and not a weakness. Instead, it should be viewed as a demonstration of competency when one seeks help in breaking the IP cycle, rather than perpetuating the stages.

## Conclusion

It is crucial to teach medical professionals about how the impostor phenomenon affects health. Furthermore, adopting policies and practices that support well-being and cultivate a positive environment can greatly enhance the welfare of physicians and medical students. Medical programs and administrators should actively work to reduce stress and promote an inclusive and supportive learning environment, better equipping medical professionals to manage the heavy demands of clinical training. The above recommendations are provided to be of benefit for those engaged in any stage of medical training. These strategies should assist in decreasing the prevalence of IP among medical professionals and thereby enhance the effectiveness of healthcare teams. By applying these tips, educators, mentors, and professional leaders can play a crucial role in helping individuals overcome IP, fostering environments where everyone has the confidence to pursue their goals and recognize their values and capabilities.
